# PPI3D: a web server for searching, analyzing and modeling protein–protein, protein–peptide and protein–nucleic acid interactions

**DOI:** 10.1093/nar/gkae278

**Published:** 2024-04-15

**Authors:** Justas Dapkūnas, Albertas Timinskas, Kliment Olechnovič, Miglė Tomkuvienė, Česlovas Venclovas

**Affiliations:** Institute of Biotechnology, Life Sciences Center, Vilnius University, Saulėtekio av. 7, Vilnius LT-10257, Lithuania; Institute of Biotechnology, Life Sciences Center, Vilnius University, Saulėtekio av. 7, Vilnius LT-10257, Lithuania; Institute of Biotechnology, Life Sciences Center, Vilnius University, Saulėtekio av. 7, Vilnius LT-10257, Lithuania; Univ. Grenoble Alpes, CNRS, Grenoble INP, LJK, 38000 Grenoble, France; Institute of Biotechnology, Life Sciences Center, Vilnius University, Saulėtekio av. 7, Vilnius LT-10257, Lithuania; Institute of Biotechnology, Life Sciences Center, Vilnius University, Saulėtekio av. 7, Vilnius LT-10257, Lithuania

## Abstract

Structure-resolved protein interactions with other proteins, peptides and nucleic acids are key for understanding molecular mechanisms. The PPI3D web server enables researchers to query preprocessed and clustered structural data, analyze the results and make homology-based inferences for protein interactions. PPI3D offers three interaction exploration modes: (i) all interactions for proteins homologous to the query, (ii) interactions between two proteins or their homologs and (iii) interactions within a specific PDB entry. The server allows interactive analysis of the identified interactions in both summarized and detailed manner. This includes protein annotations, structures, the interface residues and the corresponding contact surface areas. In addition, users can make inferences about residues at the interaction interface for the query protein(s) from the sequence alignments and homology models. The weekly updated PPI3D database includes all the interaction interfaces and binding sites from PDB, clustered based on both protein sequence and structural similarity, yielding non-redundant datasets without loss of alternative interaction modes. Consequently, the PPI3D users avoid being flooded with redundant information, a typical situation for intensely studied proteins. Furthermore, PPI3D provides a possibility to download user-defined sets of interaction interfaces and analyze them locally. The PPI3D web server is available at https://bioinformatics.lt/ppi3d.

## Graphical Abstract

**Graphical Abstract:**
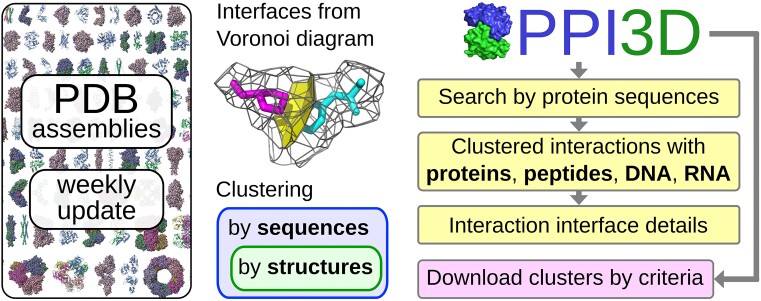

## Introduction

Proteins drive most biological processes, but they rarely act alone. Most often, proteins perform molecular functions by forming stable or temporary complexes with other proteins, peptides, nucleic acids and ligands. For comprehensive understanding of biological processes at the molecular level it is essential to know not only protein interaction partners, but also details of these interactions. This information can be obtained directly from the corresponding three-dimensional (3D) structures of protein complexes. These structures can be either determined experimentally or predicted computationally. A recent deep learning-driven breakthrough (1) resulted in accurately predicted structures for millions of individual proteins (2). However, predicting structures for protein–protein and protein–peptide complexes remains challenging (3,4), whereas prediction of protein-DNA or protein-RNA complexes is even harder (5). Therefore, the ability to utilize experimentally determined structures of protein complexes, available in the Protein Data Bank (PDB) (6) is very important for both experimentalists interested in specific proteins and computational biologists aiming at developing methods for modeling protein complexes.

The number of protein complexes in the PDB is already quite large and is growing steadily, providing a rich source for structure-resolved interaction data. However, it is not always straightforward to extract, analyze and make use of these data. One of the issues with structures determined by X-ray crystallography is how to distinguish biologically relevant interactions from those resulting from crystal packing. Another confounding issue is the redundancy of interaction data. There may be multiple PDB entries for a given protein complex, and even a single PDB entry may contain several instances of this complex. This redundancy cannot be decreased by simple sequence-based filtering, usually sufficient to obtain representative monomeric structures. The interactions within the same or a closely related complex may differ depending on conditions in which the structure was solved, the presence or absence of ligands and/or additional interacting partners. Therefore, obtaining representatives for interaction interfaces necessitates involvement of structure-based comparison. This is not a trivial endeavor in itself, because the results depend on the interface definition and the interface similarity metric.

Over the years, multiple web-based tools have been developed to address these issues and to make use of PDB interaction data for better understanding protein interactions and functions. A large family of tools, exemplified by PISA (7), *de facto* standard in PDB, EPPIC (8), ProtCID (9) and ProtCAD (10), are aiming at identification of biologically relevant protein–protein interfaces and/or biological assemblies from crystal structures. However, still there is no foolproof method against occasional erroneous assignment of biologically relevant assemblies or interfaces (11). Many other tools are dedicated to annotate and classify PDB interaction data, and use these data to transfer 3D information to homologs or infer new interactions. They all differ greatly in user interface, the range of analyses performed and output data types. Some examples include 3did (12), which links PDB interaction data to Pfam domains, HOMCOS (13), which focuses on applying interaction data for searching and template-based modeling of homologous protein complexes, and DNAproDB (14) specializing in classification and annotation of protein-DNA complexes. There also are web servers that attempt to enrich protein–protein interaction networks with 3D structures including Interactome3D (15), LEVELNET (16) and Proteo3Dnet (17).

Here, we present the updated PPI3D server, which provides a possibility to search through a non-redundant set of pairwise interactions derived from an up-to-date set of PDB biological assemblies and to analyze the obtained results in detail. In addition, PPI3D users can make homology-based inferences regarding interaction sites of their query proteins and construct template-based models. PPI3D stands out among other similar tools by the use Voronoi tessellation to derive and analyze interaction interfaces. One of the strengths of this approach is that it unambiguously defines the contribution of each residue-residue contact to the interaction interface. Furthermore, representation of contacts via contact surface areas in PPI3D enables robust structure-based clustering of interfaces and binding sites. This step is important in detecting alternative interactions, that would be lost in clustering based only on sequence similarity. Compared with the initial version of PPI3D (18), we introduced two major improvements: (i) extended the PPI3D functionality into protein–nucleic acid interactions and (ii) provided a possibility to download customizable sets of interaction interfaces. These improvements in PPI3D open up new opportunities to study interactions for protein(s) of interest and to analyze interaction interfaces in bulk.

## Materials and methods

### Analysis of structural data and definition of interaction interfaces

**Figure 1.**
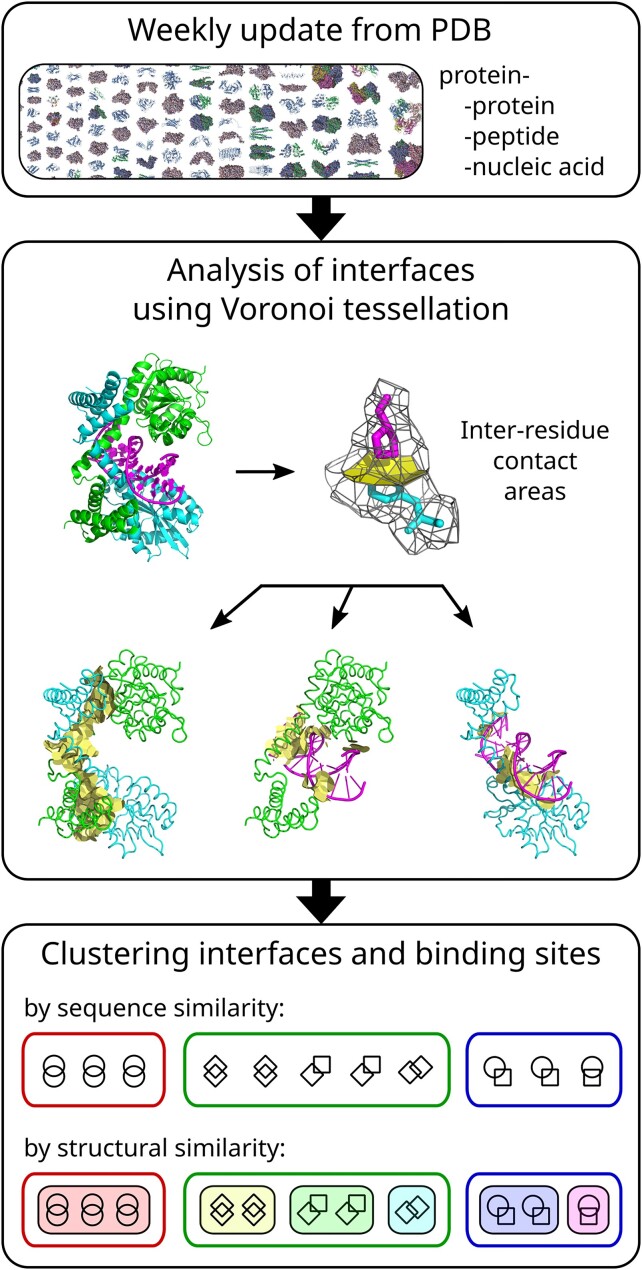
Structural data pre-processing in the PPI3D web server.

Main steps in data pre-processing by PPI3D are shown in Figure 1. The Biological Assemblies for all non-NMR structures having resolution better than 4 Å are downloaded from the PDB (6). Polypeptide chains are classified into proteins and peptides. Peptides are defined as polypeptide chains with <20 structurally-resolved residues or <40 structurally-resolved residues if more than half represent non-standard amino acids. Biological assemblies containing nucleic acids are analyzed using DSSR (19), and chains that form double-stranded DNA or RNA helices are joined into a single nucleic acid entity. Next, binary protein–protein, protein–peptide and protein–nucleic acid interactions are identified and analyzed by means of Voronoi tessellation, implemented in Voronota (20). Voronoi tessellation is a space-partitioning method. When applied to molecular structures, it assigns every atom a region of space, called Voronoi cell, which encompasses all the space points that are closer to that atom than to any other atom. Adjacent Voronoi cells share a surface, called Voronoi face, which can be viewed as geometric representation of a contact between two atoms. Contacts between atoms can be aggregated into contacts between residues (see Supplementary Data and Supplementary Figure S1 for details). The interaction interface is defined as the set of contacts between residues from different chains. The binding site is defined as the set of protein residues involved in the interaction with another entity (protein, peptide or nucleic acid). Only interfaces with the surface area over 100 Å^2^ and only unique interfaces within each PDB entry are retained for further analysis. Next, hydrogen bonds (21), disulfide bonds and salt bridges are assigned for each interface. The distributions of interface areas, numbers of inter-chain contacts and inter-chain hydrogen bonds for the interfaces in the PPI3D database are provided in Supplementary Figures S2-S4.

### Clustering of interaction interfaces and binding sites

Protein-protein interaction interfaces and protein–protein, protein–peptide, protein–nucleic acid binding sites are clustered based on protein sequence and interaction interface/binding site similarity. Initially, the protein sequences are clustered with CD-HIT (22) and for every cluster a multiple sequence alignment (MSA) is generated using L-INS-i, an accuracy-oriented MAFFT algorithm (23). MSAs are needed at a later stage to assign corresponding residues in different proteins for structure-based clustering.

Next, the protein–protein interaction interfaces are clustered by grouping interfaces where both proteins are in the same respective CD-HIT clusters. Protein binding sites are grouped by assigning the proteins of the same CD-HIT cluster to a single cluster of binding sites. These sequence-based clusters are further analyzed at the structural level by considering the interaction interface or binding site similarity, defined by the variants of CAD-score (24,25). Definition of CAD-score variants is provided in Supplementary information. To compute CAD-score values, the one-to-one correspondence between the residues in different proteins is required. Here, residues are considered to be equivalent if they are in the same column of the MSA representing a CD-HIT cluster. In case CAD-score indicates that the sequence-based clusters display structural heterogeneity, they are further split into structurally similar clusters. This sequence- and structure-based clustering procedure ensures identification of different binding sites for a given protein and/or alternative protein–protein interaction modes. Clustering of structurally similar interfaces and binding sites is done using the Taylor-Butina algorithm (26). The algorithm uses a matrix of all pairwise similarities between objects to group those objects into clusters using a provided similarity threshold (see Supplementary data for details). One of the advantages, offered by this algorithm, is a possibility to easily update clusters.

PPI3D offers the following interface clusters: (i) identical or nearly identical interface, typically representing multiple instances of the same interacting proteins or their point mutants (sequence similarity > 95%, similarity of interface contacts > 50%), (ii) highly similar interfaces usually derived from homologous protein complexes (sequence similarity > 40%, similarity of interface contacts > 50%), and (iii) similar interfaces that correspond to similar surface patches, but tolerate rearrangement of residue-residue contacts across the interface (sequence similarity > 40%, similarity of interface areas > 50%). Likewise, protein binding sites are grouped into: (i) identical or nearly identical binding sites (sequence similarity > 95%, similarity of binding site residue areas > 50%), (ii) highly similar binding sites (sequence similarity > 40%, similarity of binding site residue areas > 50%) and (iii) similar binding sites (sequence similarity > 40%, similarity of binding site areas > 50%). Detailed statistics on data reduction upon clustering is provided in Supplementary Table S1 and Supplementary Figure S5.

### Web server implementation

All the pre-processed and clustered structural data on diverse protein interactions are saved in a MySQL database. Newly released PDB entries are analyzed using the same pipeline and added to the database every week to keep in sync with the newest experimental data.

The PPI3D web server was developed using the CodeIgniter framework (https://www.codeigniter.com/). The interactive features were implemented using jQuery (https://jquery.com/). Structures are visualized interactively in a web browser using JSmol (http://jsmol.sourceforge.net/). For offline visualization, PyMOL scripts are provided. BLAST applications are used from the BLAST + package (27). Homology modeling is done by MODELLER (28). Structure alignments are generated using TM-align (29).

## PPI3D web server description

### Web server workflow

#### Input

**Figure 2.**
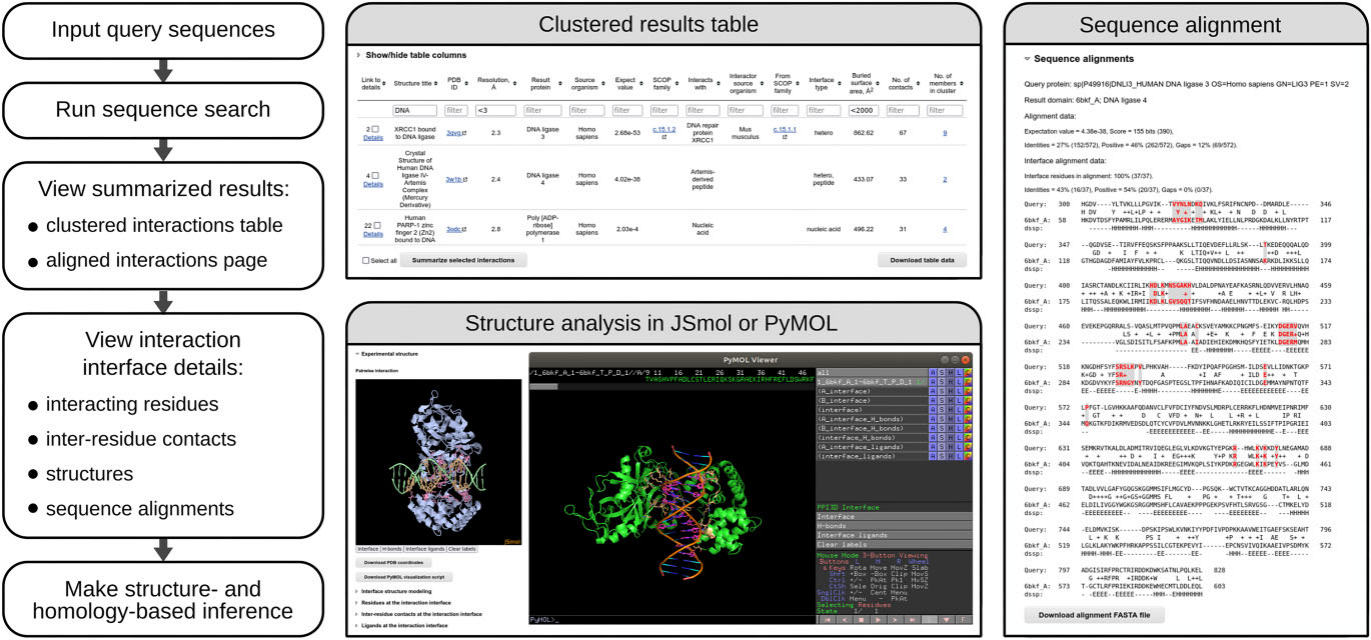
PPI3D search and analysis workflow with examples of output features.

A typical workflow of the PPI3D web server is illustrated in Figure 2. The input into the server are protein sequences, UniProt accession codes, or PDB IDs. In the latter case, the server retrieves and displays all the binary interactions in a single PDB entry. Protein sequences (or corresponding UniProt codes) are used to find structural data on interactions of the query proteins and/or their homologs. Sequence-based search has two modes: (i) ‘single-sequence’ search to query interactions for a given protein with any proteins, peptides and nucleic acids and (ii) ‘two-sequences’ search to identify only protein–protein interactions between the first and the second proteins or their homologs.

#### Query processing

When the user inputs the query sequence(s), the PPI3D server searches in its database of protein sequences associated with structural interaction data using either BLAST or PSI-BLAST (27,30). The PPI3D job with the BLAST option runs very fast, because the search is performed directly in the PPI3D database of protein sequences, but detects only close homologs. If the PSI-BLAST option is chosen, the server first generates a Position-Specific Scoring Matrix (PSSM) for the query sequence by searching iteratively in a local copy of the NCBI non-redundant sequence database (https://ftp.ncbi.nlm.nih.gov/blast/db/), and then uses the resulting PSSM to search the PPI3D database. In this case a search takes more time to complete, but more distant homologs can be detected.

#### Output

The results on the interaction data are provided in a hierarchical manner. Initially, only the summarized information is shown, simply displaying how many pairwise interactions of different types (protein–protein, protein–peptide, protein–nucleic acid) are found for each of the query proteins (or protein pairs). By default, the output represents the most stringent clustering option, but the clustering stringency can be interactively adjusted. Next, the user might choose to analyze the lists of identified interactions.

The lists of identified interaction interfaces or binding sites are displayed in a table, containing protein annotations, calculated interface properties, BLAST *E*-values (if search by protein sequences was used), and cluster sizes. Sometimes the redundancy reduction by clustering might be insufficient, therefore, PPI3D allows selecting multiple results and summarizing them. This is done by displaying the alignment of selected results to the query sequence. The interacting residues are highlighted in the resulting multiple sequence alignment, making it easy to see whether there is a similar pattern of interacting residues at least in some of the interfaces or binding sites. To further inspect the similarities or differences between the search results, one can align selected structures with TM-align (29) and then visualize them in JSmol.

For even more in-depth analysis, PPI3D provides a very detailed page for every search result, showing the interaction properties, the structures of the binary interaction alone and in the context of the entire biological assembly, and tables listing interface residues and inter-residue contacts. The interacting residues for the user's query proteins can be inferred from the displayed highlighted sequence alignments or from generated homology models (31). The structures can be inspected using JSmol directly in the browser or could be downloaded as scripts for visualization in PyMOL. The users, interested in the analysis of interactions not only at the residue, but also at the atomic level, may choose to automatically transfer the structures to the VoroContacts server (32). VoroContacts makes it possible to analyze not only the entire interface, but also user-defined subsets of interface contacts that may be further filtered by various attributes.

### Use case examples

In this section we provide several examples, illustrating how PPI3D could be used to search and analyze diverse protein interactions.

#### Exploring bacterial DNA sliding clamp interactions

**Figure 3.**
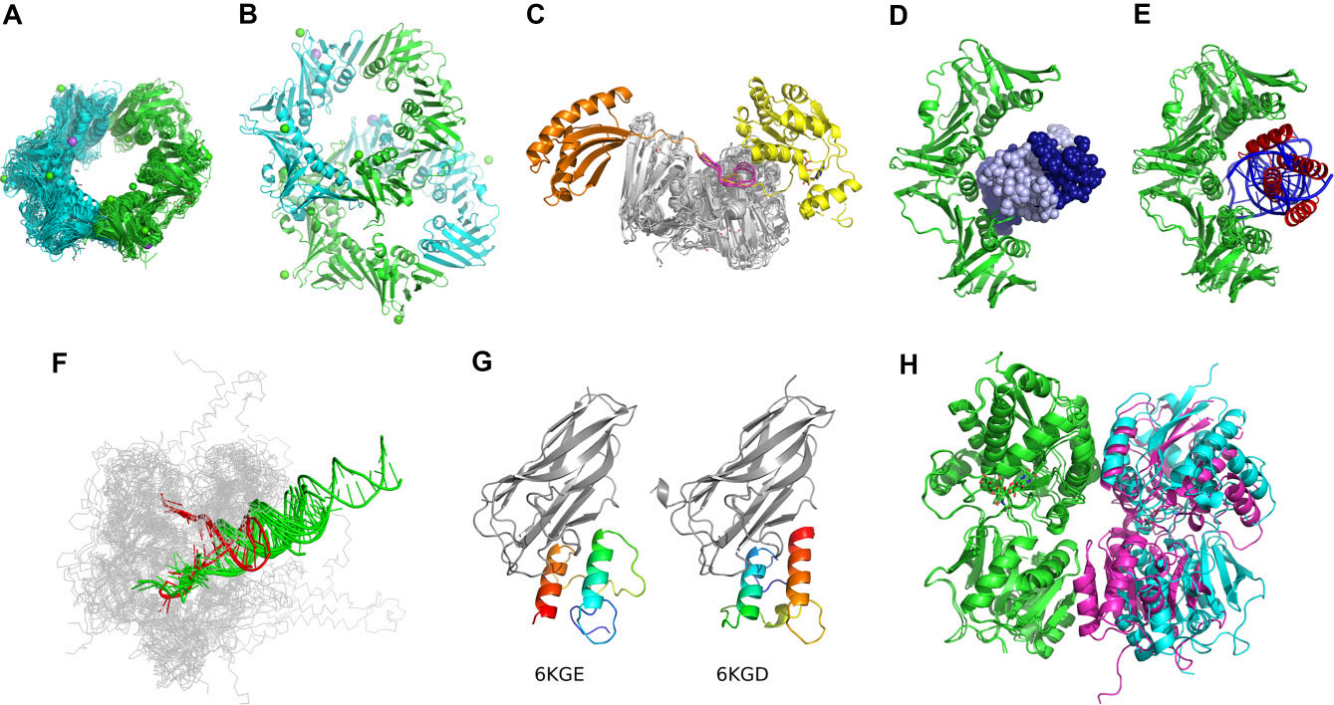
Examples of analyses using PPI3D. (**A**) Homodimers of DNA sliding clamp homologs listed in Supplementary Table S2; (**B**) two stacked rings of sliding clamps in PDB entry 8DT6; (**C**) heteromeric interactions of DNA sliding clamps with protein linear motifs or peptides (sliding clamp, gray; DNA polymerase IV (PDB: 1UNN), orange; DnaA regulatory inactivator Hda (PDB: 5X06), yellow; 7 peptides, magenta); (**D**) DNA (dark and light blue) binds differently to sliding clamp subunits (green) (PDB: 3BEP); (**E**) viral replication inhibitors (red, PDB: 7EVP) bind to the same site of sliding clamp as DNA (blue, PDB: 3BEP); (**F**) DNA polymerases (grey) show distinct DNA binding modes in the polymerization (green) and proofreading (red) modes; (**G**) dual binding modes of cohesin-dockerin interaction; dockerin (colored) bound to cohesin (gray) is flipped by 180 degrees in different modes; (**H**) extracellular domains of human GABA_B_ receptor heterodimer in active (magenta and green, PDB: 4MS3) and inactive (cyan and green, PDB: 4MR8) forms; heterodimers are superimposed by aligning subunit 2 (green).

DNA sliding clamp functions by encircling the DNA helix and serving as a mobile platform, to which various proteins involved in DNA transactions can bind. To identify interactions that the clamp participates in, we used the PPI3D ‘single-sequence’ search mode. A BLAST search with *E. coli* DNA sliding clamp (Uniprot AC: P0A988) revealed a large number of interactions, involving proteins, peptides and DNA (Supplementary Table S2). Most homomeric protein–protein interactions fall into two large clusters representing *E. coli* and *M. tuberculosis* proteins. Summarizing clamp binding sites both at the sequence level and by superimposing cluster representatives revealed that they all bind another subunit to form a closed ring (Figure 3A). An exception is the DNA sliding clamp from *Elizabethkingia anopheles* (PDB: 8DT6), which has alternative interfaces in addition to the consensus interface (Figure 3B). The PPI3D data indicate that the alternative interfaces, resulting from two stacked rings, are outliers. Surprisingly, both PDBePISA (7) and EPPIC (8) consider the two stacked rings to represent a biological assembly. However, in such assembly the central cavity of each ring is blocked and the DNA cannot be threaded through the sliding clamp suggesting that this type of arrangement is the result of crystal packing rather than a biologically relevant structure.

Over 100 detected protein–peptide binding sites within sliding clamps can be grouped into just 7 clusters. Structure superposition of the DNA sliding clamp subunits further demonstrates that all these binding sites correspond to the same structural region. Proteins also tend to bind to the same region using linear motifs (Figure 3C). This is indeed expected as it is known that different proteins bind to the DNA sliding clamp within a structurally conserved pocket (33,34).

There is only one PDB entry that has a clamp bound to DNA (PDB: 3BEP) (35). PPI3D data show that DNA is bound asymmetrically to the clamp (Figure 3D). The detailed binding site data reveal that R24, one of the two residues important for the clamp function (35), binds DNA in both subunits, whereas Q149 only binds DNA in one of the subunits. Interestingly, viral protein Gp168 that inhibits bacterial DNA replication also binds to the same site of the sliding clamp (Figure 3E) (36).

#### Detecting different DNA binding modes in DNA polymerases

DNA polymerases are essential enzymes that catalyze DNA synthesis during replication and reparation. The most widespread B-family DNA polymerases have DNA polymerase and 3′–5′ exonuclease activities that are located in different domains. To investigate how these enzymes interact with DNA, we queried PPI3D with the sequence of an unexplored DNA polymerase from *Halorubrum halophilum* (RefSeq: WP_050032690.1) using PSI-BLAST. The server found 271 protein–nucleic acid binding sites, grouped into 33 clusters, in polymerases from eukaryotes, archaea, bacteria, and viruses. We selected 11 representative binding sites with largest surface areas (>1800 Å^2^) (Supplementary Table S3). Using the ‘Summarize selected interactions’ feature we aligned their structures on the *Thermococcus sp*. DNA polymerase solved in the replicative state (PDB: 5OMV) (37). Superposition revealed that polymerases bind DNA in two different modes, corresponding to DNA synthesis and proofreading (Figure 3F) (38,39).

#### Detecting alternative protein–protein interactions

Among the vast diversity of protein–protein interactions, alternative binding modes are occasionally observed (40). Therefore, searches for interactions in databases clustered only by protein sequences might miss some of the interfaces. Since PPI3D clusters the interaction interfaces not only by sequence, but also by structure, it allows identification of the alternative binding modes.

One of the well-known dual binding protein pairs is cohesion and dockerin, domains found in a cellulosome, an enzymatic complex of anaerobic cellulolytic microorganisms (Figure 3G). The biological significance of this dual binding mode is still unknown (41), but it was discovered that it can be regulated by pH (42). After a PSI-BLAST search in PPI3D using the sequences of PDB entry 6KGE, we found 32 structures that can be clustered differently. A more stringent clustering (sequence identity 40%, similarity of interface residue contacts > 50%) produced 17 clusters, whereas a more lenient clustering that disregards specific residue-residue contacts (sequence identity 40%, similarity of interface areas > 50%) produced only 12 clusters (Supplementary Table S4).

Changes of protein binding modes can also occur upon ligand binding. For example, the agonist binding causes large conformational changes in heterodimeric human GABA_B_ receptor, inducing formation of additional inter-subunit contacts and doubling the total interface area (from ∼700 to ∼1400 Å^2^) (Figure 3H) (43). PPI3D clustering recognized these two interfaces as distinct clusters.

### Downloading of the PPI3D data

The PPI3D user interface offers interactive analysis of diverse protein interactions. All the tables can be sorted by different properties and filtered using text, regular expressions or numerical values. The structures can be visualized in JSmol. Yet, in some cases it may be more convenient to analyze the data offline. Therefore, the data displayed in the PPI3D web server including tables, structures, and sequence alignments can be downloaded for local use.

In addition to the analysis of interaction data for specific protein(s), PPI3D also provides a possibility to download other user-defined subsets of the clustered structural protein interaction data in bulk. The users can select the PPI3D data subsets according to different criteria and download the data in tabular format as well as coordinate files. These data sets might be useful for detailed investigation of protein interactions at scale or for training machine learning models.

## Discussion

PPI3D web server offers a user-friendly environment for searching and analyzing structure-resolved protein-centered interactions. PPI3D may be especially helpful if no interaction data are available for the protein(s) of interest. Sequence searches in the ‘single-sequence’ mode may help to infer putative interaction partners based on the identified structure-resolved homologs bound to other proteins, peptides or nucleic acids. Likewise, the identified interactions in the ‘two-sequences’ search mode may suggest that two query proteins interact. In both cases, these initial hypotheses can be further explored at the residue level using both sequence alignments with the detected structural homologs and template-based models.

The server has already proved useful in both experimental (44,45) and computational studies (46,47). PPI3D also helped our group to achieve top results in the protein assemblies modeling category in recent CASP and CAPRI experiments (48,49). In the AlphaFold era template-based modeling is becoming less important (50), but the ability to quickly test hypotheses on whether specific residues might be involved in binding with the help of homology models and to survey the broader structural context for the query protein(s) remains very useful. In contrast to protein–protein complexes, the accurate structure prediction of protein–nucleic acid complexes is still largely refractory. Therefore, the ability of PPI3D to provide homology-based inferences related to protein-DNA or protein-RNA interactions is highly relevant.

Within the ecosystem of tools dedicated to the analysis of structure-resolved data on biomolecular interactions (7–10,12–17), PPI3D features a unique set of capabilities. PPI3D uses precomputed non-redundant structural data that are updated weekly to keep in sync with PDB. In contrast, most other servers, except for those directly associated with PDB (7,13), are usually based on PDB data that are several months or even several years old. PPI3D offers sequence-based searches that can be tuned to detect either only close or also distant interacting homologs. The user interface allows interactive analysis of diverse interactions for the proteins of interest within the common framework both at the sequence and structure levels. The analysis may range from the most general data regarding the identified interfaces/binding sites down to the properties of individual residue-residue contacts. To the best of our knowledge, PPI3D is the only server that uses rigorous Voronoi tessellation-based methodology for the clustering and analysis of interactions. A newly introduced option to download all the data on interaction interfaces might be useful for large-scale analyses. It can also be beneficial for providing well-defined up-to-date datasets for training and testing machine learning methods for predicting structures or properties of macromolecular complexes. The information about how interaction interfaces are clustered on both sequence and structure levels may be especially important for defining training/validation/testing data splits. To conclude, the PPI3D web server might be useful for both experimental and computational research involving protein interactions.

## Supplementary Material

gkae278_Supplemental_File

## Data Availability

The PPI3D server and downloadable data on protein interaction interfaces are freely available at https://bioinformatics.lt/ppi3d.
