# Supplementary material for: PPI3D: a web server for searching, analyzing and modeling protein–protein, protein–peptide and protein–nucleic acid interactions
Source: Nucleic Acids Res. 2024 Apr 15;52(W1):W264–71. doi: 10.1093/nar/gkae278 (PMC11223826; doi:10.1093/nar/gkae278)
Supplement: gkae278_Supplemental_File [file gkae278_supplemental_file.pdf]

PPI3D: a web server for searching, analyzing and modeling protein-protein, protein-peptide and protein-nucleic acid interactions

Supplementary data

Justas Dapkūnas[1], Albertas Timinskas[1], Kliment Olechnovič[1,2], Miglė Tomkuvienė[1] and Česlovas Venclovas[1]*

[1]Institute of Biotechnology, Life Sciences Center, Vilnius University, Saulėtekio av. 7, Vilnius LT-10257, Lithuania
[2]Univ. Grenoble Alpes, CNRS, Grenoble INP, LJK, 38000 Grenoble, France

*To whom correspondence should be addressed. Tel: +370-5-223-4368; Email: ceslovas.venclovas@bti.vu.lt

Contents

1. Voronoi tessellation-derived representations of contacts 2
2. Properties of interaction interfaces in the PPI3D database 3
3. Defining structural similarity of protein interaction interfaces and binding sites 6
   3.1 Interaction interfaces 6
   3.2 Binding sites 7
4. Clustering of interaction interfaces and binding sites using the Taylor-Butina algorithm 8
5. Reduction of data redundancy by clustering 10
6. Examples of PPI3D search results 12

1. Voronoi tessellation-derived representations of contacts

Voronoi tessellation is a subdivision of space into regions called Voronoi cells. Every Voronoi cell is defined for a central generating object, for example a disk in 2D or a ball in 3D. Every Voronoi cell contains all the space points that are closer to its generating object than to other objects. Some points are equidistant to two generating objects – they form the boundaries between adjacent Voronoi cells, that is, edges between 2D Voronoi cells or faces between 3D Voronoi cells. Those boundaries are representations of contacts between generating objects, for example, atoms. The 2D and 3D examples in Supplementary Figure S1 illustrate derivation of contact representations from Voronoi tessellation.

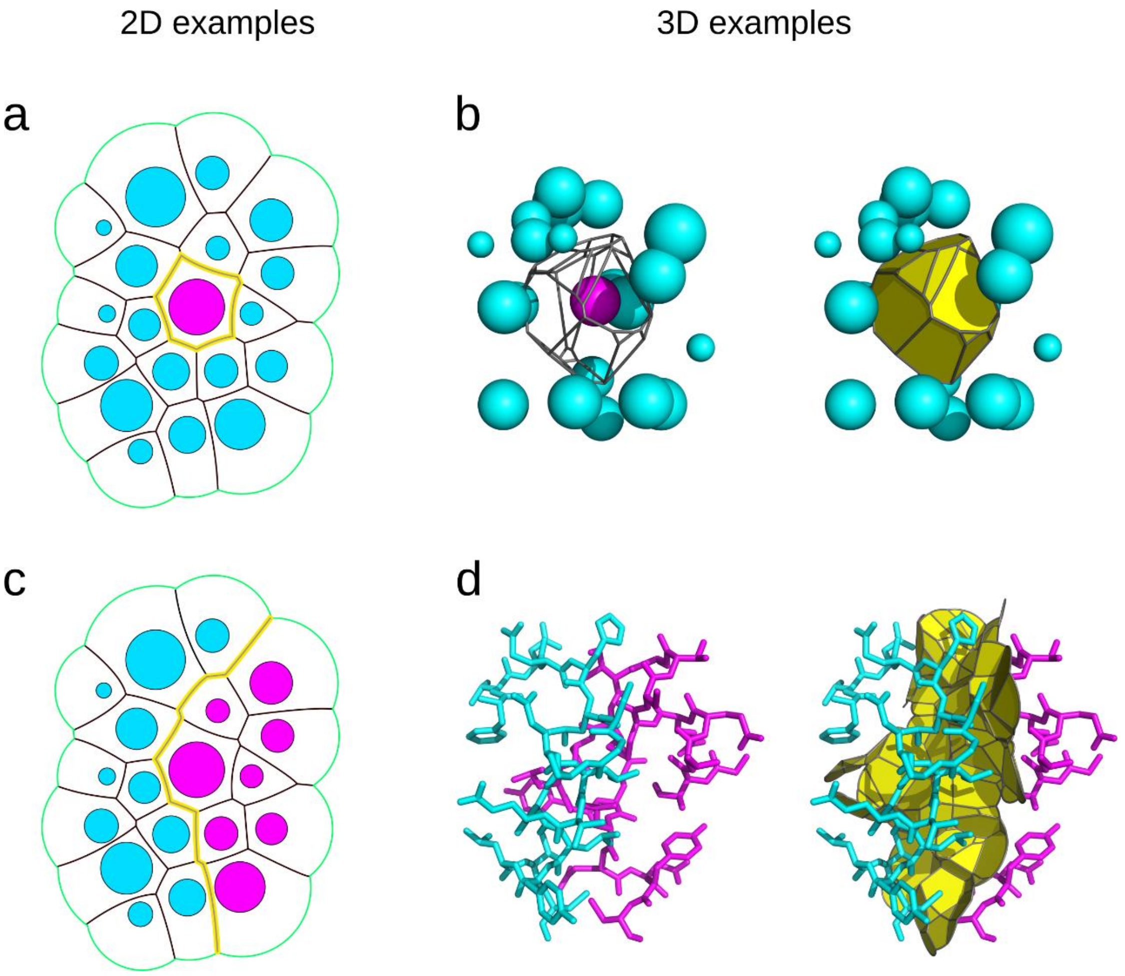

Supplementary Figure S1. Derivation of contact representation from Voronoi tessellation. (a) Boundaries (edges) of a single 2D Voronoi cell highlighted in yellow, shown inside the Voronoi tessellation (constrained inside the probe-defined green boundary); (b) Boundaries (edges and faces) of a single 3D Voronoi cell shown together with the neighboring 3D balls that correspond to the adjacent Voronoi cells; (c) Interface between two groups of 2D disks highlighted in yellow, defined as the set of boundaries (Voronoi cell edges) between the 2D Voronoi cells of disks from different groups; (d) Interface between two chains of atoms in an insulin molecular structure (PDB: 6VER) defined as the set of Voronoi cell faces between the 3D Voronoi cells of atomic balls from different chains. The 3D Voronoi cells are constrained inside the solvent-accessible surface of the molecular structure.

2. Properties of interaction interfaces in the PPI3D database

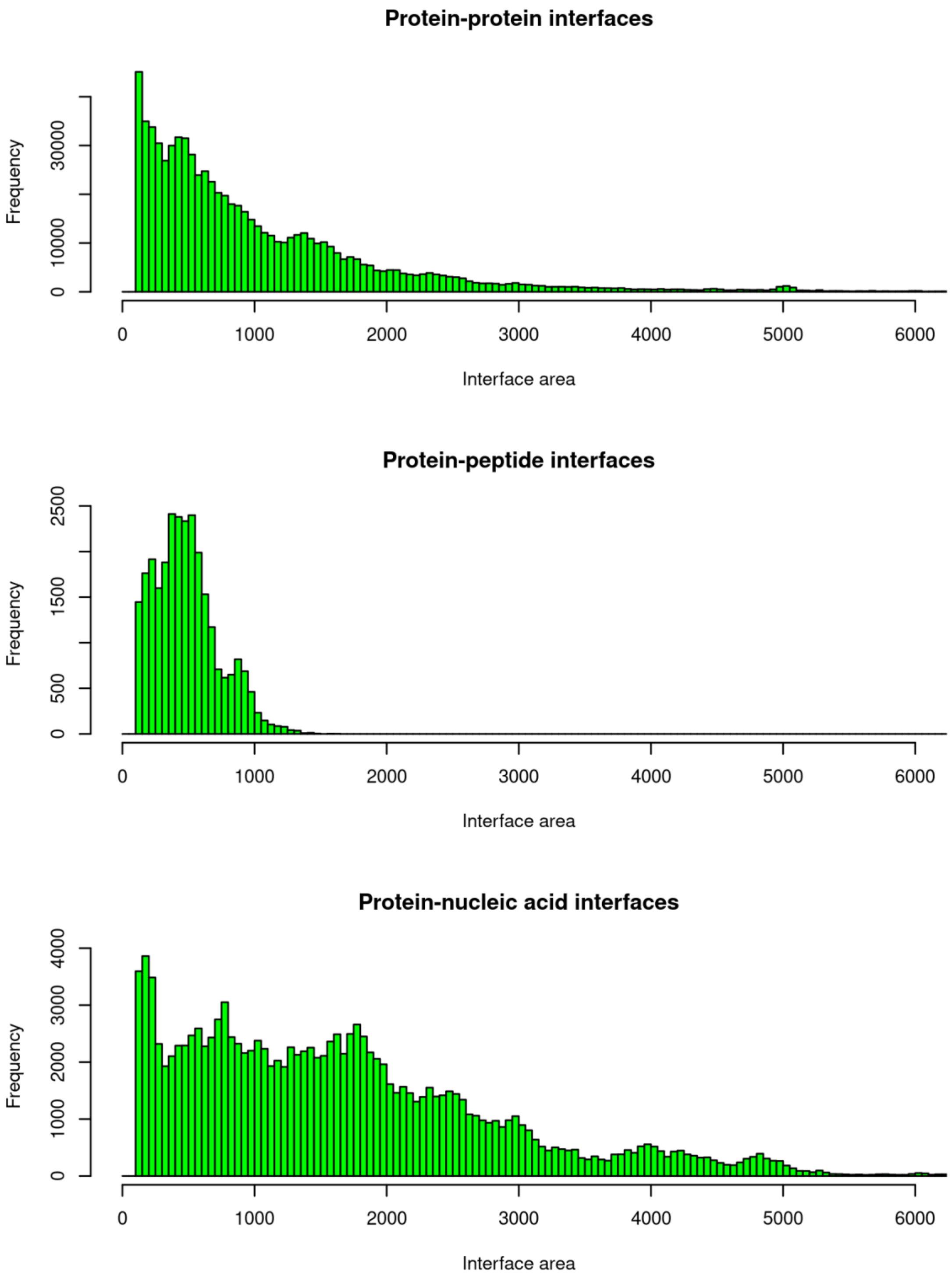

Supplementary Figure S2. Histograms of the areas (in Å^2) of protein-protein, protein-peptide and protein-nucleic acid interfaces in PPI3D database (based on the PDB data as of March 13th, 2024).

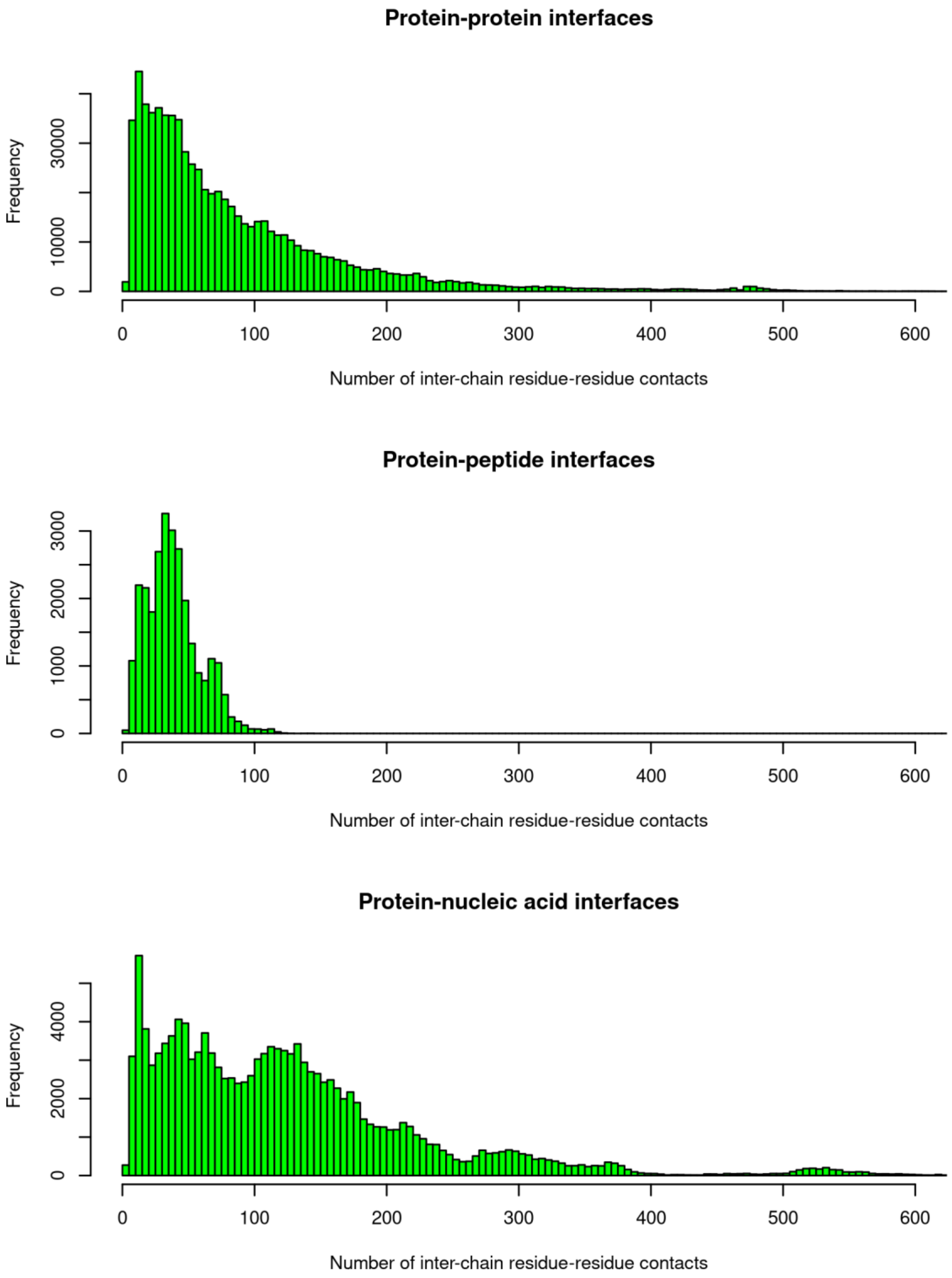

Supplementary Figure S3. Histograms of the number of inter-chain residue-residue contacts in the protein-protein, protein-peptide and protein-nucleic acid interfaces in PPI3D database (based on the PDB data as of March 13th, 2024).

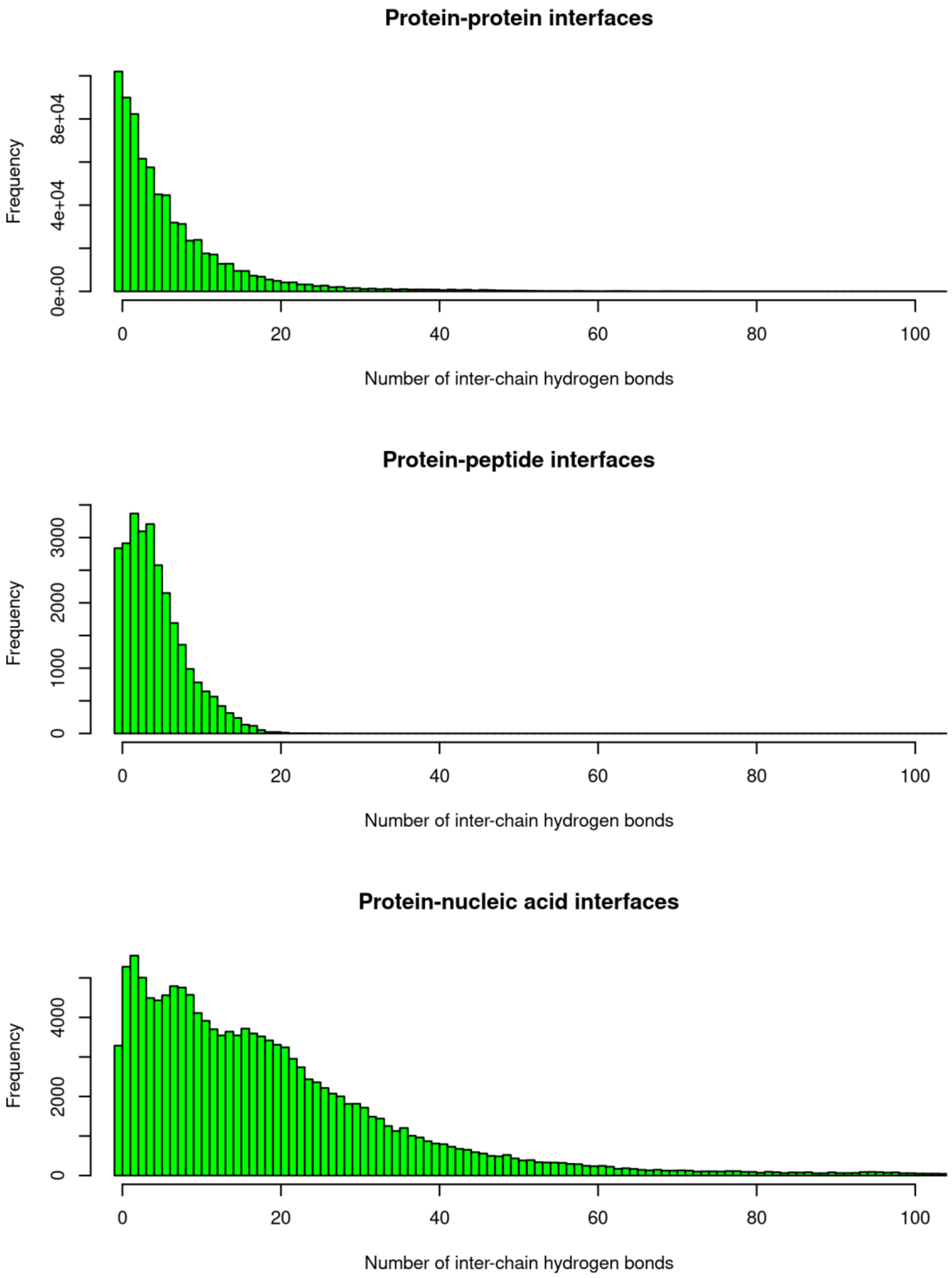

Supplementary Figure S4. Histograms of the number of inter-chain hydrogen bonds in the protein-protein, protein-peptide and protein-nucleic acid interfaces in PPI3D database (based on the PDB data as of March 13th, 2024).

3. Defining structural similarity of protein interaction interfaces and binding sites

3.1 Interaction interfaces

Similarity of interface residue contacts

Given two dimeric structures S^1 and S^2, let G^1 denote the set of all the pairs of residues (i,j) such that i and j belong to different subunits (chains) and have a non-zero contact area $S^1_{(i,j)}$ in the first (reference) structure. Then, for every residue pair $(i,j) \in G^1$, the corresponding contact area $S^2_{(i,j)}$ in the S^2 structure is calculated. $S^2_{(i,j)}$ is assigned zero if there is no contact between residues i and j in the S^2 or if either residue (i or j) is missing from the S^2. The interface CAD-score for S^1 and S^2 is then defined as:

$$\text{CAD-score}^{\text{iface}}(S^1, S^2) = 1 - \frac{\sum_{(i,j)\in G^1} \min(|S^1_{(i,j)} - S^2_{(i,j)}|, S^1_{(i,j)})}{\sum_{(i,j)\in G^1} S^1_{(i,j)}} \qquad (1)$$

Values of Equation 1 are always within the [0,1] range. If S^1 and S^2 structures are identical, $\text{CAD-score}^{\text{iface}}(S^1, S^2) = 1$. At the other extreme, if S^1 and S^2 have no common contacts, $\text{CAD-score}^{\text{iface}}(S^1, S^2) = 0$.

CAD-score values are not symmetric, that is, in general,

$\text{CAD-score}^{\text{iface}}(S^1, S^2) \neq \text{CAD-score}^{\text{iface}}(S^2, S^1)$.

In PPI3D, the symmetric interface similarity score is defined as:

$$\text{CAD-score}^{\text{iface}}_{\text{sym}}(S^1, S^2) = \min(\text{CAD-score}^{\text{iface}}(S^1, S^2), \text{CAD-score}^{\text{iface}}(S^2, S^1)) \qquad (2)$$

Similarity of interface areas

Less stringent measure of interface similarity can be defined using total sums of interface contact areas in S^1 and S^2:

$$\text{CAD-score}^{\text{iface-area}}(S^1, S^2) = \min\left(1, \frac{\sum_{(i,j)\in G^1} S^2_{(i,j)}}{\sum_{(i,j)\in G^1} S^1_{(i,j)}}\right) \qquad (3)$$

This similarity measure quantifies how closely the interface in S^2 corresponds to the same surface patch in S^1 without paying attention to the exact contribution by individual residue-residue contacts. The symmetric version of this measure is defined as the minimum of the asymmetric score values, similarly to Equation 2:

$$\text{CAD-score}^{\text{iface-area}}_{\text{sym}}(S^1, S^2) = \min(\text{CAD-score}^{\text{iface-area}}(S^1, S^2), \text{CAD-score}^{\text{iface-area}}(S^2, S^1)) \quad (4)$$

3.2 Binding sites

Similarity of binding site residue areas

It is possible to quantify how each interface residue is exposed to the other chain by summing the relevant contact areas. Let I_a^1 denote the set of interface residues of the subunit a in the S^1 complex. For a specific residue $i \in I_a^1$, the exposure value in S^1 is $S_i^1 = \sum_{(i,j)\in G^1} S_{(i,j)}^1$. The set of S_i^1 values for all $i \in I_a^1$ describes the binding site of chain a in the S^1 structure. The set of S_i^2 values for all $i \in I_a^1$ is defined in the same way, but using the S^2 interface contacts, that is, $S_i^2 = \sum_{(i,j)\in G^2} S_{(i,j)}^2$. Then, the similarity score of the S^1 and the S^2 binding sites in chain a is defined as:

$$\text{CAD-score}^{\text{site}}(S^1, S^2, a) = 1 - \frac{\sum_{i\in I_a^1} \min(|S_i^1 - S_i^2|, S_i^1)}{\sum_{i\in I_a^1} S_i^1} \quad (5)$$

The symmetric version of this measure is defined as the minimum of the asymmetric score values, similarly to Equations 2 and 4.

Similarity of binding site areas

A less stringent measure of the binding site similarity can be defined using total sums of binding site residue areas in S^1 and S^2:

$$\text{CAD-score}^{\text{site-area}}(S^1, S^2, a) = \min\left(1, \frac{\sum_{i\in I_a^1} S_i^2}{\sum_{i\in I_a^1} S_i^1}\right) \quad (6)$$

This similarity measure quantifies how closely the binding site in S^2 corresponds to the same surface patch in S^1 without paying attention to the exact contribution by individual residues. The symmetric version of this measure is defined as the minimum of the asymmetric score values, similarly to Equations 2 and 4.

4. Clustering of interaction interfaces and binding sites using the Taylor-Butina algorithm

Interaction interfaces and binding sites are first clustered according to the similarity of protein sequences, and then the resulting clusters are split according to structure similarity, defined by CAD-score. The clustering according to structure similarity is performed using the Taylor-Butina algorithm. This algorithm is based on exclusion spheres. Having the similarity matrix and the desired similarity threshold, first the number of neighbors (items that are more similar than the selected threshold) is calculated for every item. The items that have no neighbors are marked as singletons, and the items having neighbors are clusterable items. The clusterable item having the largest number of neighbors is set to be the center of the first cluster. This center item and all its neighbors are assigned to the first cluster and are subsequently removed from the dataset. In the resulting smaller dataset the items having no neighbors are marked as false singletons (i.e., items, that had neighbors in the full dataset, but not after excluding some clusters) and clusterable items. The process is repeated until no clusterable items are left. In the end, false singletons are assigned to the most similar cluster.

The algorithm is illustrated more formally below in Algorithm S1.

Algorithm S1. Taylor-Butina clustering.

```
Input: similarity matrix, similarity threshold
Output: clusters
Find neighbors given similarity matrix and similarity threshold;
Find singletons and define clusterable items;
Mark singletons as true singletons;
while there are clusterable items:
        Find item with largest number of neighbors and make it cluster centroid;
        Create new cluster from cluster centroid and its neighbors;
        Remove cluster centroid and its neighbors from dataset;
        Find singletons and define clusterable items;
        Mark new singletons as false singletons;
end while
for every false singleton:
        Find maximum similarity to cluster representatives;
        Assign false singleton to this cluster;
end for
Create clusters from true singletons;
```

When updating the PPI3D database with new PDB data, new interaction interfaces or binding sites are first assigned to sequence-based clusters. Then CAD-score similarity values are computed between

each new interface/binding site and representatives of structure-based clusters. The new item is assigned to the cluster of maximum similarity, if it is higher than threshold, or forms a new cluster if no clusters with similarity exceeding the desired threshold are found (Algorithm S2).

Algorithm S2. Update clusters derived using Taylor-Butina clustering.

```
Input: clusters, similarity threshold, new items
Output: updated clusters
for every new item:
    Calculate CAD-scores with cluster representatives;
    Find cluster having maximum CAD-score;
    if maximum CAD-score value > similarity threshold then
        Append item to cluster with maximum CAD-score;
    else
        Create new cluster from item;
    end if
end for
```

5. Reduction of data redundancy by clustering

Supplementary Table S1. Summary of clustered protein interaction data in PPI3D database (based on the PDB data as of March 13th, 2024).

**Protein-protein interaction interfaces**	
All interfaces	744507
Clustered at sequence similarity > 95%	
By sequence similarity only	79862
By sequence similarity and interface residue contacts similarity > 50%	129423
Clustered at sequence similarity > 40%	
By sequence similarity only	49881
By sequence similarity and interface residue contacts similarity > 50%	103539
By sequence similarity and interface area similarity > 50%	78982
**Protein-protein binding sites**	
All binding sites	1489014
Clustered at sequence similarity > 95%	
By sequence similarity only	55435
By sequence similarity and residue interface areas similarity > 50%	185423
Clustered at sequence similarity > 40%	
By sequence similarity only	31022
By sequence similarity and residue interface areas similarity > 50%	136633
By sequence similarity and binding site area similarity > 50%	110852
**Protein-peptide binding sites**	
All binding sites	27537
Clustered at sequence similarity > 95%	
By sequence similarity only	4134
By sequence similarity and residue interface areas similarity > 50%	5712
Clustered at sequence similarity > 40%	
By sequence similarity only	2431
By sequence similarity and residue interface areas similarity > 50%	4440
By sequence similarity and binding site area similarity > 50%	3645
**Protein-nucleic acid binding sites**	
All binding sites	136431
Clustered at sequence similarity > 95%	
By sequence similarity only	8042
By sequence similarity and residue interface areas similarity > 50%	12687
Clustered at sequence similarity > 40%	
By sequence similarity only	4636
By sequence similarity and residue interface areas similarity > 50%	8994
By sequence similarity and binding site area similarity > 50%	7145

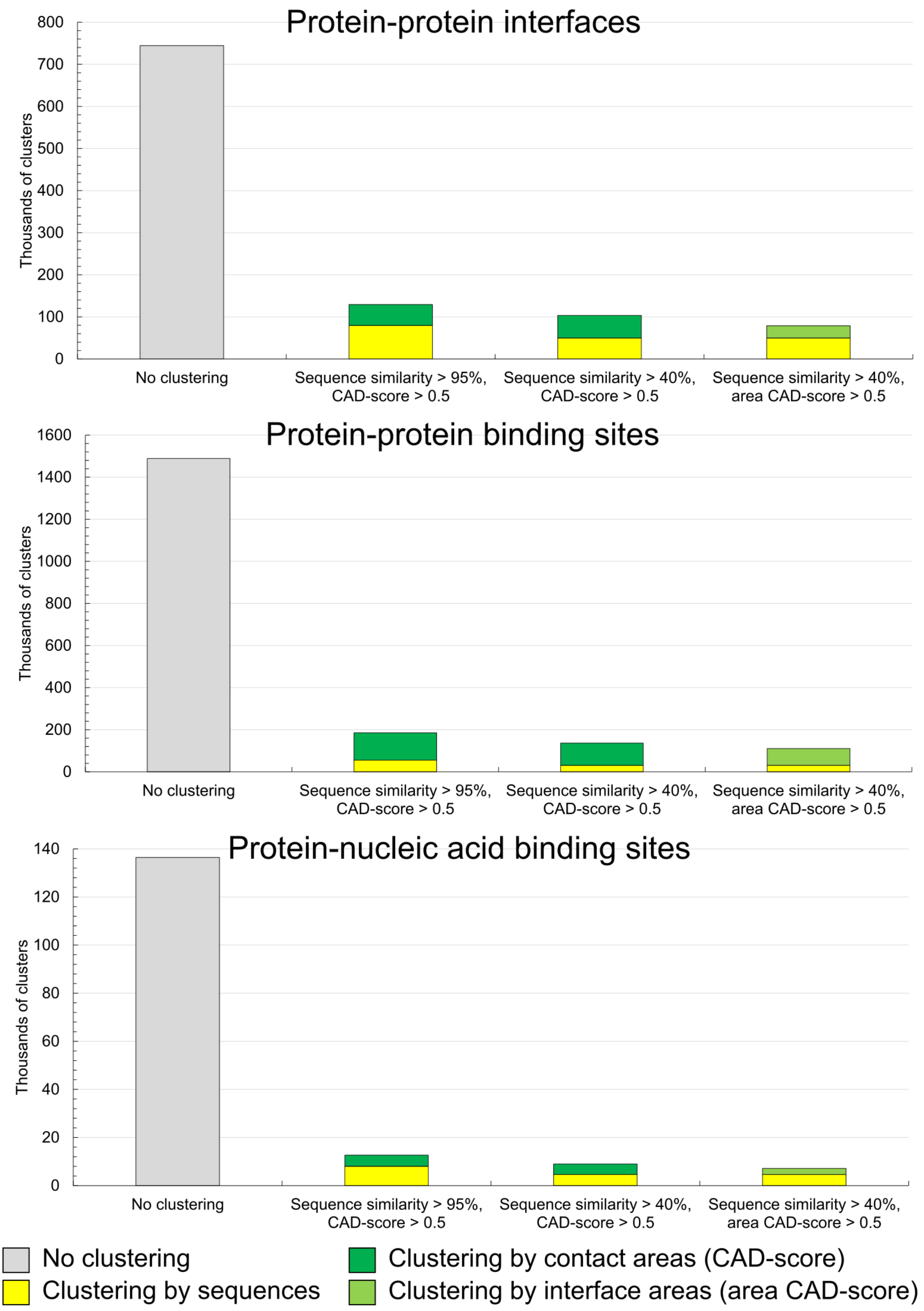

Supplementary Figure S5. Reducing protein interaction data redundancy in the PPI3D database by clustering interactions according to protein sequence and interaction interface/binding site similarity.

6. Examples of PPI3D search results

Supplementary Table S2. Clustered results of PPI3D BLAST search for homologs of *E. coli* DNA sliding clamp (protein-peptide interactions are denoted using gray background).

PDB ID	Result protein	Source organism	Interacts with	Buried surface area, Å²	No. of members in cluster
Homomeric interactions					
7azf	Beta sliding clamp	Escherichia coli 2-427-07_S4_C3	Beta sliding clamp	1297.51	182
6pth	Beta sliding clamp	Pseudomonas aeruginosa PAO1	Beta sliding clamp	1183.65	2
5wce	DNA polymerase III subunit beta	Caulobacter vibrioides CB15	DNA polymerase III subunit beta	1201.63	12
6man	Beta sliding clamp	Rickettsia bellii RML369-C	Beta sliding clamp	1085.63	14
4tr6	DNA polymerase III subunit beta	Bacillus subtilis	DNA polymerase III subunit beta	1195.54	8
1vpk	DNA polymerase III, beta subunit	Thermotoga maritima	DNA polymerase III, beta subunit	1195	2
7evp	Beta sliding clamp	Staphylococcus aureus	Beta sliding clamp	1170.32	2
7ybd	Beta sliding clamp	Clostridioides difficile	Beta sliding clamp	1068.77	2
8dt6	Beta sliding clamp	Elizabethkingia anophelis NUHP1	Beta sliding clamp	278.78	1
8dt6	Beta sliding clamp	Elizabethkingia anophelis NUHP1	Beta sliding clamp	1098.7	4
8dt6	Beta sliding clamp	Elizabethkingia anophelis NUHP1	Beta sliding clamp	179.36	1
8dt6	Beta sliding clamp	Elizabethkingia anophelis NUHP1	Beta sliding clamp	1429.75	2
8dt6	Beta sliding clamp	Elizabethkingia anophelis NUHP1	Beta sliding clamp	179.36	1
8dt6	Beta sliding clamp	Elizabethkingia anophelis NUHP1	Beta sliding clamp	278.78	1
4trt	DNA polymerase III subunit beta	Deinococcus radiodurans	DNA polymerase III subunit beta	1060.78	2
6fvo	Beta sliding clamp	Mycobacterium tuberculosis CDC1551	Beta sliding clamp	1125.27	46
3t0p	DNA polymerase III, beta subunit	Agathobacter rectalis	DNA polymerase III, beta subunit	1185.77	2
6dj8	Beta sliding clamp	Borreliella burgdorferi B31	Beta sliding clamp	1385.19	2
5g4q	DNA POLYMERASE III SUBUNIT BETA	Helicobacter pylori 26695	DNA POLYMERASE III SUBUNIT BETA	1053.99	16
Heteromeric interactions					
7azf	Beta sliding clamp	Escherichia coli 2-427-07_S4_C3	Peptide 8	394.15	87
6p81	Ubiquitin-like protein SMT3,Beta sliding clamp	Klebsiella pneumoniae IS22	Griselimycin	581.32	2
1unn	DNA POLYMERASE III BETA SUBUNIT	Escherichia coli	DNA POLYMERASE IV	328.36	2
1unn	DNA POLYMERASE III BETA SUBUNIT	Escherichia coli	DNA POLYMERASE IV	1140.28	2
5x06	DNA polymerase III subunit beta	Escherichia coli O157:H7	DnaA regulatory inactivator Hda	1147.23	4
1jqj	DNA polymerase III, beta chain	Escherichia coli	DNA polymerase III, delta subunit	1258.27	2
6ptr	Beta sliding clamp	Bartonella birtlesii LL-WM9	ACE-MVA-MP8-NZC-LEU-MP8-LEU-MVA-PRO-MLU-GLY	608.63	2

PDB ID	Result protein	Source organism	Interacts with	Buried surface area, Å²	No. of members in cluster
6djk	Beta sliding clamp	Rickettsia typhi str. Wilmington	ACE-MVA-MP8-NZC-LEU-MP8-LEU-MVA-PRO-MLU-GLY	526.27	5
7evp	Beta sliding clamp	Staphylococcus aureus	Sliding clamp inhibitor	194.19	2
7evp	Beta sliding clamp	Staphylococcus aureus	Sliding clamp inhibitor	503.28	2
6fvo	Beta sliding clamp	Mycobacterium tuberculosis CDC1551	P7 peptide	553.44	32
6dj8	Beta sliding clamp	Borreliella burgdorferi B31	Natural product peptide	558.88	2
5frq	DNA POLYMERASE III SUBUNIT BETA	Helicobacter pylori 26695	DNA LIGASE	521.6	1
Protein-nucleic acid interactions					
3bep	DNA polymerase III subunit beta	Escherichia coli	DNA	357.08	1
3bep	DNA polymerase III subunit beta	Escherichia coli	DNA	197.85	1

Supplementary Table S3. Representative protein-nucleic acid interactions of the homologs of *Halorubrum halophilum* DNA polymerase having interfaces larger than 1800 Å^2. Complexes corresponding to the proofreading mode are highlighted in gray.

**Structure title**	**PDB ID**	**Result protein**	**Source organism**	**Expect value**	**Interacts with**	**Buried surface area, Å^2**
Ternary complex of 9N DNA polymerase in the replicative state with two metal ions in the active site	5omv	DNA polymerase	Thermococcus sp. 9oN-7	1.15e-140	DNA template/DNA primer	1802.42
Crystal structure of DNA polymerase from Thermococcus gorgonarius in complex with hypoxanthine-containing DNA	2xhb	DNA POLYMERAS E	Thermococcus gorgonarius	2.69e-140	HYPOXANTH INE-CONTAINING DNA	1960.56
Pyrococcus abyssi B family DNA polymerase bound to a dsDNA, in edition mode	4flt	DNA polymerase 1	Pyrococcus abyssi GE5	6.17e-140	Template strand/Primer strand	2201.77
Processive human polymerase delta holoenzyme	6tny	DNA polymerase delta catalytic subunit	Homo sapiens	1.88e-100	DNA template/DNA primer	1937.12
Ternary complex of DNA polymerase delta	3iay	DNA polymerase delta catalytic subunit	Saccharomyces cerevisiae	9.26e-97	Nucleic acid	2085.03
Cryo-EM structure of S. cerevisiae DNA polymerase alpha-primase complex in the DNA elongation state	8fok	DNA polymerase	Saccharomyces cerevisiae	1.99e-87	template DNA/RNA-DNA chimeric primer	1831.61
Crystal structure of E.coli Pol II-normal DNA-dGTP ternary complex	3maq	DNA polymerase II	Escherichia coli K-12	3.38e-78	DNA	2005.1
HSV1 polymerase ternary complex with dsDNA and PNU-183792	7luf	DNA polymerase	Human alphaherpesvirus 1	1.05e-37	DNA	1917
Closed ternary complex of an RB69 gp43 fingers domain mutant complexed with an acyclic GMP terminated primer template pair and phosphonoformic acid.	3kd5	DNA polymerase	Escherichia phage RB69	1.24e-29	DNA	1975.4
The structure of MPXV polymerase holoenzyme in replicating state	8hg1	DNA polymerase	Monkeypox virus	1.30e-23	DNA (38-MER)/DNA (25-MER)	2028.45
The crystal structure of M644G variant of DNA Pol Epsilon containing dCTP in the polymerase active site	8b6k	DNA polymerase epsilon catalytic subunit A	Saccharomyces cerevisiae	1.25e-16	Template DNA sequence/ Primer DNA sequence	1895.34

Supplementary Table S4. *Clostridium acetobutylicum* cohesin-dockerin interologs having interface area over 500 Å^2. Alternative interaction interfaces that are merged into larger clusters upon the interface area-based clustering are highlighted in gray

PDB ID	Protein 1	Protein 1 source organism	Expect value 1	Protein 2	Expect value 2	Buried surface area, Å²
6kge	Probably cellulosomal scaffolding protein, secreted cellulose-binding and cohesin domain	Clostridium acetobutylicum ATCC 824	1.12e-45	And cellulose-binding endoglucanase family 9 CelL ortholog dockerin domain	6.20e-18	673.46
6kgd	Probably cellulosomal scaffolding protein, secreted cellulose-binding and cohesin domain	Clostridium acetobutylicum ATCC 824	1.12e-45	And cellulose-binding endoglucanase family 9 CelL ortholog dockerin domain	1.10e-17	670.15
4fl4	Cellulosome anchoring protein cohesin region	Acetivibrio thermocellus	2.98e-21	Glycoside hydrolase family 9	4.30e-12	825.15
3ul4	Cellulosome-anchoring protein	Acetivibrio thermocellus	9.67e-22	Cellulosome enzyme, dockerin type I	2.21e-09	798.03
4dh2	Cellulosome anchoring protein cohesin region	Acetivibrio thermocellus	3.25e-19	Dockerin type 1	2.31e-08	802.21
2ccl	CELLULOSOMAL SCAFFOLDING PROTEIN A	Acetivibrio thermocellus	1.40e-13	ENDO-1,4-BETA-XYLANASE Y	7.40e-08	813.48
1ohz	Cohesin domain	Acetivibrio thermocellus	2.55e-13	Endo-1,4-beta-xylanase Y	3.49e-07	755.26
5nrk	Endoglucanase	Acetivibrio cellulolyticus	2.05e-24	DocCel5: Type I dockerin repeat domain from A. cellulolyticus family 5 endoglucanase WP_010249057 S15I, I16N mutant	7.45e-07	779.07
4uyq	CELLULOSOMAL SCAFFOLDIN ANCHORING PROTEIN C	Acetivibrio cellulolyticus	3.61e-23	CELLULOSOMAL SCAFFOLDIN ADAPTOR PROTEIN B	1.33e-06	876.21
5nrm	Endoglucanase	Acetivibrio cellulolyticus	8.64e-24	DocCel5: Type I dockerin repeat domain from A. cellulolyticus family 5 endoglucanase WP_010249057 S51I, L52N mutant	1.51e-06	772.03
2vn6	SCAFFOLDING PROTEIN	Ruminiclostridium cellulolyticum	6.38e-27	ENDOGLUCANASE A	1.84e-06	739.59
4uyp	CELLULOSOMAL SCAFFOLDIN ANCHORING PROTEIN C	Acetivibrio cellulolyticus	3.61e-23	CELLULOSOMAL SCAFFOLDIN ADAPTOR PROTEIN B	2.19e-06	785.74
2vn5	SCAFFOLDING PROTEIN	Ruminiclostridium cellulolyticum	6.38e-27	ENDOGLUCANASE A	7.95e-06	767.97
5lxv	Scaffoldin C	Ruminococcus flavefaciens FD-1	2.66e-05	Carbohydrate-binding protein WP_009985128	2.91e-08	1376.25
